# T-Cell Mediated Inflammation in Postmenopausal Osteoporosis

**DOI:** 10.3389/fimmu.2021.687551

**Published:** 2021-06-30

**Authors:** Di Wu, Anna Cline-Smith, Elena Shashkova, Ajit Perla, Aditya Katyal, Rajeev Aurora

**Affiliations:** Department of Molecular Microbiology and Immunology, Saint Louis University School of Medicine, St. Louis, MO, United States

**Keywords:** T cell, postmenopausal osteoporosis, estrogen loss, osteoimmunology, chronic inflammation

## Abstract

Osteoporosis is the most prevalent metabolic bone disease that affects half the women in the sixth and seventh decade of life. Osteoporosis is characterized by uncoupled bone resorption that leads to low bone mass, compromised microarchitecture and structural deterioration that increases the likelihood of fracture with minimal trauma, known as fragility fractures. Several factors contribute to osteoporosis in men and women. In women, menopause – the cessation of ovarian function, is one of the leading causes of primary osteoporosis. Over the past three decades there has been growing appreciation that the adaptive immune system plays a fundamental role in the development of postmenopausal osteoporosis, both in humans and in mouse models. In this review, we highlight recent data on the interactions between T cells and the skeletal system in the context of postmenopausal osteoporosis. Finally, we review recent studies on the interventions to ameliorate osteoporosis.

## Introduction

A great achievement of modern medicine is the increased lifespan of the human population. Unfortunately, the comorbidities of aging have created a large economic and health burden on society. The current challenge is to improve the healthspan and thus to reduce the burden. Osteoporosis is the most prevalent metabolic bone disease that affects half the women and one third of men, typically, in the sixth and seventh decade of life (1, 2). Osteoporosis is characterized by uncoupled bone resorption that leads to low bone mass, compromised microarchitecture and structural deterioration that increases the likelihood of fracture with minimal trauma. These fragility fractures lead to disproportionally high mortality rate and a drastic decline in quality of life for those affected.

Bone remodeling occurs th0roughout life and is a coordinated process to repair microfractures and maintain bone mass. Imbalances in the bone remodeling process underscore the pathophysiology of osteoporosis. Bone remodeling is a tightly coupled: resorption precedes formation and the amount of bone formed is balanced with the amount resorbed. Remodeling can be initiated by hormonal, environmental and nutritional factors (3). The major cell types involved in bone remodeling are bone resorbing osteoclasts (OC) and bone forming osteoblasts (OB). Over the last decade the bone-embedded osteocytes (Ocy) have also emerged as a key regulators. OC are multinucleated cells from the monocytic lineage whose differentiation depends on receptor activator of NF-κB (RANK) and its ligand (RANKL). OB differentiate from the mesenchymal stem cell (MSC) lineage and is regulated by several signaling pathways such as WNT/β-catenin and BMP. During remodeling the OC and OB form the bone remodeling unit (BRU). Ocy are stellate like cells enclosed within mineralized bone. They serve as mechanosensors within the bone and play a key regulatory role in bone homeostasis, directing and coordinating repair by regulating the BRU.

It was recognized nearly eight decades ago that involutional osteoporosis in postmenopausal women is mediated by loss of estrogen (E2) (4). The mechanism for how E_2_ loss leads to increased bone resorption has remained, despite intense focus of investigations (5). Decreased calcium absorption (6, 7), decline in renal function (8) and impaired vitamin D metabolism (9, 10) with aging and menopause. Over the past three decades there has been growing appreciation that the adaptive immune system plays a fundamental role in the development of postmenopausal osteoporosis (PMOP), both in humans and in mouse models. The recognition that T-cell derived cytokines affect bone has given rise to the field of osteoimmunology, a word was first coined in 2000 by Arron and Choi (11). There have been major advances in our understanding of the pro-resorptive effects of pro-inflammatory cytokine, in particular TNFα and interleukine IL-17A made by T cell. In this review, we highlight recent data on the interactions between T cells and the skeletal system in the context of PMOP. We also review recent studies on the interventions to ameliorate osteoporosis, with insights into immunomodulatory options. Finally, we highlight some questions that still remain unanswered.

## Postmenopausal Osteoporosis

While dietary, lifestyle and other factors impact bone health (12, 13), in general there are two main reasons for the decline of bone mass. The skeletal system grows rapidly postnatally and through puberty and peak bone mass is attained by mid to late 20s (14). Both men and women gradually loss bone mass as they age (15) and the rate of loss varies by anatomical site (16). In addition to aging, loss of sex hormones and in particular estrogen (E_2_) contribute to skeletal homeostasis (5, 17, 18).

Sex hormones increase during puberty and are maintained during reproductive age. While testosterone (T) decreases linearly with age in men, women experience a sharp decline in E_2_ at menopause. Menopause, is the cessation of ovarian function, is one of the leading causes of primary osteoporosis. Early studies suggested that E_2_ directly regulates OC (19–22) and OB (23, 24) and its loss results in long lived OC and impaired OB leading to uncoupled bone resorption (25). Accordingly, PMOP has been traditionally regarded as an endocrinal, E_2_ deficiency mediated disease. While epidemiological observation suggest that E_2_ loss is responsible for osteoporosis in both sexes, the mechanism in males remains unclear. The role of estrogen and androgens on the bone have been extensively reviewed previously (25–27). In the next section, we will focus on the role of the immune system in the pathogenesis of PMOP.

## The Crosstalk Between the Bone and the Immune System

**Figure 1:**
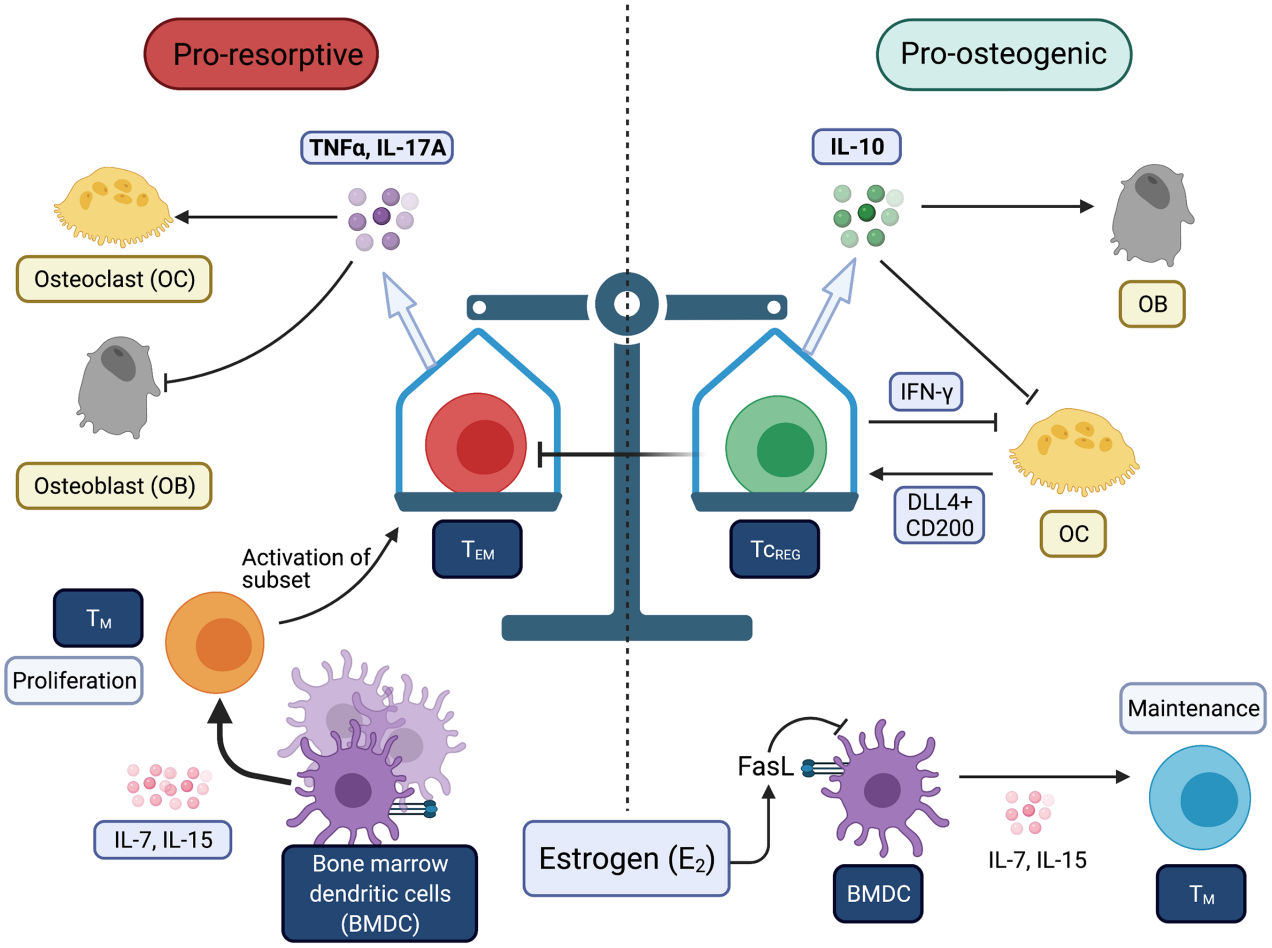
Balance between immunogenic and tolergenic states is linked with resorptive and osteogenic states of the bone. Chronic inflammation may be derived by increases in effector T-cells (T_EFF_) and effector memory T-cells (T_EM_). Bone is very sensitive to TNFα and IL-17A produced by T-cells. Estrogen (E_2_) prevents the conversion of T_M_ to T_EM_. Tolerogenic T-cells (i.e., T_REG_ and Tc_REG_) promote bone formation through direct and indirect mechanisms. All Figures were created with (BioRender.com).

In the past few decades, evidence has emerged supporting the notion that E_2_ loss promotes persistent inflammation that promote osteoporosis and perhaps other comorbidities. The mechanistic studies for linking E_2_ loss at menopause and activation of the T cells has come from ovariectomy (OVX) of rodents and key outcomes have been validated in human studies. In this section, we highlight recent advances in our understanding on how T cells and proinflammatory cytokines, namely TNFα and IL-17A, contribute to the pathogenesis of PMOP (**Figure 1**).

### Inflammation Tips the Balance in Favor of Bone Resorption Through Osteoclasts

Takayanagi et al. were the first to report the bone-immune cross talk, demonstrating that T-cell produced IFN-γ can inhibit RANKL signaling during OC differentiation (28). Because Th1 are major producers of IFN-γ, inflammatory bone loss was thought as a Th1 mediated pathology. It was later demonstrated that Th17 cells are key drivers of bone erosion (29) and IL-17A is a potent promoter of bone destruction, particularly in the context of autoimmune pathologies (30–32). TNFα has also been shown to directly act on OC and its precursors in synergy with RANKL to promote osteoclastogenesis (33–36). Bone appears to be sensitive to T-cell derived cytokines even at distal anatomical sites. For instance, decline in bone mass is observed in patients with chronic HIV (37, 38), Hepatitis B and C (39) infections. Hepatic viruses also affect conversion of vitamin D3 to the metabolically active form calcitriol because they infect hepatocytes and affect calcium absorption in addition to increased IL-17A production by T-cells (40, 41). Increased prevalence of fracture are also observed in patients with rheumatoid arthritis (RA), inflammatory bowel disease (IBD) and chronic obstructive pulmonary disease (COPD) (42–45). The local cytokine milieu can contextually promote or protect against bone loss and the mechanism for how TNFα and IL-17A favor bone resorption *via* OC have been reviewed extensively (46, 47).

**Figure 2:**
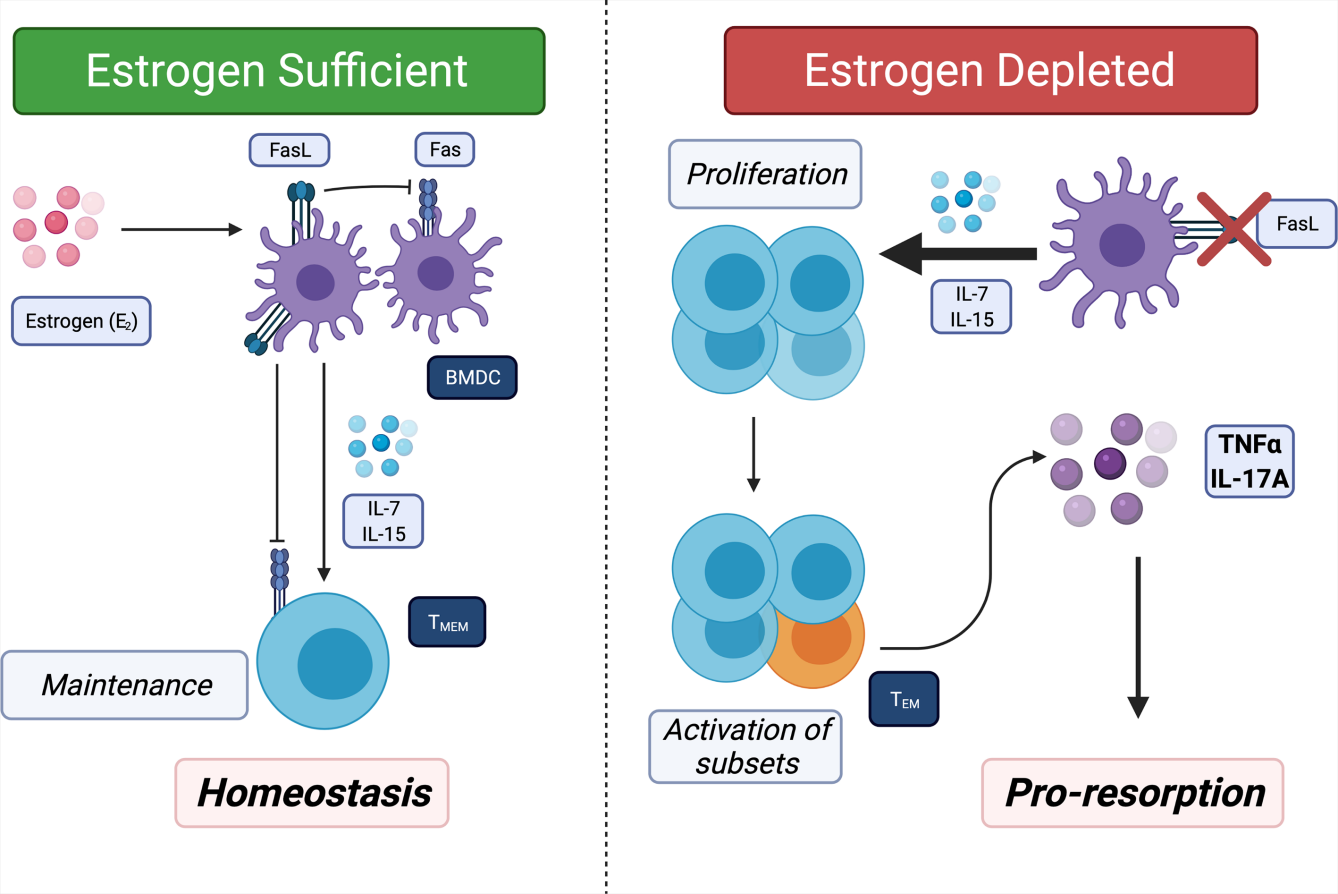
Estrogen (E_2_) regulates bone marrow resident memory T-cells (T_BRM_) homeostasis and E_2_ loss promotes conversion of T_BRM_ to T_EM_. T_M_ migrate to and take up long-term residence in the bone marrow. *Left panel*: Bone marrow resident dendritic cells (BMDC) secrete IL-7, IL-15 or both to promote survival of T_M_. In E_2_ replete females, BMDC have a short lifespan because E_2_ induces FasL in the BMDC. In addition, IL-15 induces Fas in proliferating T_M_ in response to IL-7 and IL-15 thus maintain a homeostatic pool of T_BRM_. *Right*: In absence of E_2_, Fas ligand (FasL) is no longer induced leading to increased lifespans of BMDC and high concentrations of IL-7 and IL-15. In the presence of high IL-7 and IL-15 and absence of FasL, all T_M_ proliferate and a subset (~5 to 10%) produce TNFα and IL-17A, which then promotes bone resorption and also limits bone formation.

In the past decade, several studies showed that the immune system and inflammation play a critical pathogenic role in uncoupled bone loss in the context of E_2_ loss (30, 48–52). OVX of sexually mature mice that were T-cell deficient showed decreased bone loss, demonstrating that T-cells are required for promoting bone resorption (53–57). Recently, our lab has described a new pathway where E_2_ loss leads to chronic low-grade production of the proinflammatory cytokines TNFα and IL-17 by converting memory T-cells (T_M_) to effector T_M_ (T_EM_). We showed that there us increased levels of IL-7 and IL-15. Both these cytokines are produced by bone marrow dendritic cells (BMDCs). E_2_ induces Fas ligand and apoptosis of BMDC and also T_M_. In the absence of E_2_, the BMDC become long-lived, which leads to higher IL-7 and IL-15 and to antigen-independent activation of a subset of T_M_ to produce TNFα and IL-17A (58) (**Figure 2**). The canonical activation of T_M_ and subsequent conversion to T_EM_ are antigen-dependent (59), thus the activation observed by E_2_-loss does not follow the established paradigm. OVX of IL15Rα^ΔT-cells^ mice, where T_M_ cannot convert into T_EM_, did not result in bone loss (58). Our results uncovered another aspect of how E_2_ is anti-inflammatory, as it maintains T_M_ homeostasis and limits their conversion to T_EM_ in the absence of antigen.

The gut microbiome (GMB) plays an important role in regulating bone mass. A number of studies have shown an association between GBM and bone health in both animal models (60, 61) and in humans (13). Germfree (GF) mice have increased bone mass compared to conventionally raised (CONV-R) mice, and restoration of GBM normalized bone mass in GF mice (62). E_2_ loss increases gut permeability (63–65), which leads to increased priming and activation of inflammation in the gut mucosa, leading to the generation Th17 cells (66). GF mice do not lose bone post-OVX, and probiotics can prevent OVX-induced bone loss (67, 68). Recent studies have suggested GMB produce microbial metabolites that have regulatory function on distal organs, including the bone. GMB derived butyrate, polyamines and short-chain fatty acids have been shown to induce regulatory T cell (T_REG_) generation in the colon and be able to directly regulate the BRU (69–71). Thus, there appear to be several mechanisms by which GMB modulate bone health: first, GMB produce metabolites that directly modulate bone mass. Second GBM induce Th17 cell to promote bone loss or T_REG_ to limit bone loss. Finally, GBM not only induce Th17 cells but many of these effector T-cells become T_M_. A subset of T_M_ migrate to the bone marrow to become bone marrow resident T_M_ (72). Thus prior exposures of pathogens and commensals are encoded in the T_M_. This may explain (at the population level) why only about half of postmenopausal women develop osteoporosis. Women who have more exposure to Th17 inducing microbes through their life would have a larger pool of T_M_ that convert to T_EM_ that produce IL-17 and TNFα postmenopause.

### How Does Inflammation or Resolution of Inflammation Regulate Bone Mass?

The field has primarily focused on the effect of inflammation on OC as discussed above. How inflammation restrains bone formation is less well studied. Under coupled bone remodeling conditions, the increase in resorption is accompanied by recruitment of mesenchymal stromal cells (MSC) and their conversion to OB. However, in the presence of inflammatory cytokine (i.e., TNFα, IL-17 and IL-1β) this process appears to be impaired. Thus, bone formation lags behind bone resorption. Next, we discuss how proinflammatory cytokines effects the osteolineage.

Early *in vitro* culture studies showed that TNFα inhibited MSC to OB transition by regulating RUNX2 expression, a master transcription factor that commits MSC into osteogenic pathway. TNFα also targets Osterix (OSX; SP7) expression, a key transcription necessary for osteoblast maturation (73–75). Osteoblastogenesis is sensitive to glucose levels (76) and OB differentiation is regulated *via* mTOR pathway (77–80). While not clearly established in OB, there is precedence that TNFα regulates cellular metabolism in adipocytes and muscles cells (81–83). Since these three cell types all originate from MSC *via* different developmental pathway, it is likely that TNFα targets mTOR complexes in OB to alter cellular metabolism. Indeed, *in vitro* evidence demonstrated that TNFα can modulate autophagy and apoptosis *via* NF-κB signaling in OB (84–86) both of which are controlled by mTOR. While these results are controversial, there are reports indicating that IL-17A is able to affect MSC to OB differentiation as well as mature OB function and is summarized in a recent review (47). Understanding the effects of TNFα and IL-17A on OB will provide further insight into the imbalance between bone resorption and bone formation.

The effect of inflammation on Ocy is largely unknown. Ocy are regulators of bone homeostasis (87). Ocy produce RANKL that predominantly regulates osteoclastogenesis during remodeling (88, 89). In the presence of TNFα a much lower concentration of RANKL is needed to initiate osteoclastogenesis (90). Ablation of RANKL in Ocy *via* Dmp1-Cre protected against vertebral bone loss (91). TNFα and IL-17A can target Ocy to produce RANKL and thus contribute to increased resorption (92, 93). Furthermore, IL-17A can target Ocy to increase osteogenic differentiation of MSC in cooperation with OB (94). All evidence taken together, suggest that Ocy are at the center of BRU balance and regulate bone resorption and formation according to biological needs. OVX induced Ocy apoptosis (95) possibly *via* TNFα, IL-17A or both, suggesting that inflammation regulates bone health not just through OC or OB. Interestingly during lactation, where there is E_2_ loss triggers the same BMDC induced uncoupled bone resorption as OVX, Ocy have been shown to undergo a process called osteocytic osteolysis that promotes bone resorption to release calcium from cortical bone (96). Further investigation is need to understand the role Ocy play in PMOP.

It is clear that the skeletal system is exquisitely sensitive to chronic inflammation suggesting that the skeletal system is “a canary in the coal mine” – a sensor of overall persistent inflammatory burden. All currently available data is consistent with the observation that PMOP is mediated by inflammation. Specifically, E_2_ loss induces the conversion of bone marrow resident T_M_ to T_EM_ that secrete TNFα and IL-17A. These cytokines promote osteoclastogenesis and bone resorption and most likely also limit bone formation. However, the effect of TNFα and IL-17A on MSC, mature OB or Ocy is much less well understood. Future therapies for the treatment of PMOP should also address the underlying inflammation, which we will discuss in the following section.

## Therapeutic Interventions

A number of therapies have been developed to treat osteoporosis in postmenopausal women. The traditional therapies fall into two classes: anti-resorptive and bone anabolics. Each has been used independently and in some clinical trials also used in combination.

### Antiresorptive

The most commonly prescribed medication for osteoporosis are antiresorptives, notably bisphosphonates and denosumab. It has been reported that antiresorptives can interact with the immune system. Bisphosphonates have been shown to boost B-cell function and promote humoral immunity (97). Denosumab has been associated with increased risks of infection (98) and more recently, being investigated for oncology alongside immune checkpoint inhibitors (99). One drawback with this class of medications are potential severe adverse effects. Osteonecrosis of the jaw (ONJ) is observed in 1-3% of patients on anti-resorptive therapies with certain predisposing factors (i.e., after tooth extraction or in people with type 2 diabetes) (100–102). Atypical femoral fractures have also been reported in patients on bisphosphonates (103) while denosumab discontinuation have been associated with higher risk of vertebral fractures (104). Another disadvantage of anti-resorptive treatment is that there is a specific window where they are most effective. In addition, inhibition of bone resorption prevents bone remodeling and repair leading to effete bone that fractures from minimal trauma (105). As a result, while the patient may maintain BMD, it does not reflect that whether they have improved bone quality.

### Anabolics

The second class of therapies are bone anabolics. A commonly used bone anabolic is teriparatide, derived from parathyroid hormone (PTH) (106). More recently romosozumab that targets sclerostin (product of the SOST gene) (107) has been approved as a bone anabolic biologic. Neutralization of sclerostin increases OB numbers and simultaneously suppresses bone resorption thus promoting bone formation. However, due to adaptive changes in bone and potential adverse effects with prolonged use, bone anabolic therapies are limited in their use (108–110), particularly in special populations (111). Antiresorptive therapies (RANKL blockade or bisphosphonates) in postmenopausal women did not increase bone mass in the SHOTZ clinical trial (112, 113) indicating that deficit in OB activity remains. Remarkably, while bone anabolic therapy improve bone mineral density, bone microarchitecture did not improve unless both antiresorptive and anabolic agents were combined (114–116). These results suggest that efficacy is obtained by using both antiresorptive (brakes on OC) and anabolics as accelerator. Since sclerostin is primarily (but not exclusively) produced by Ocy, the action and mechanism of action on Ocy remains to be understood.

All current therapies target the cells of the BRU, either to suppress resorption or to promote bone formation. Furthermore, the current therapies have shortcomings and adverse effects with prolonged use necessitating drug holidays (117).

### Immunomodulatory Options

Therapeutic option that target the immune system has recently gained interested as treatment option for PMOP. Given that a number of inflammatory conditions lead to bone destruction, inhibition of specific cytokine signaling have also been used to disrupt the cell-cell signaling and to protect against bone destruction. Blockade of TNFα (118) and IL-17A (119) in mice have shown to prevent OVX-induced bone loss. Etanercept (anti-TNFα) have been used to treat PMOP patients and showed decreases in serum markers of bone resorption (120), which warrant further investigation whether it is safer and better than current therapies (121). A recent study showed that neutralization of IL-17A induces compensatory increase of other Th17 cytokines, including IL-17F, IL-22 and GM-CSF (122). While secukinumab (anti-IL-17A) has not been evaluated for the treatment of osteoporosis, this finding highlights the complex nature of targeting cytokines in preventing bone loss.

Probiotic supplementation can be considered as an immunomodulatory therapy, given the role of GMB in regulating bone health as mentioned in this review. Bone loss in OVX mice can be prevented with supplementation of probiotics (68). While randomized controlled trial were conducted (123) and showed reduction in bone loss in particular with *Lactobacillus reuteri* (124), these results should be interpreted with caution and further studies are needed to evaluate the benefits of probiotics.

**Figure 3:**
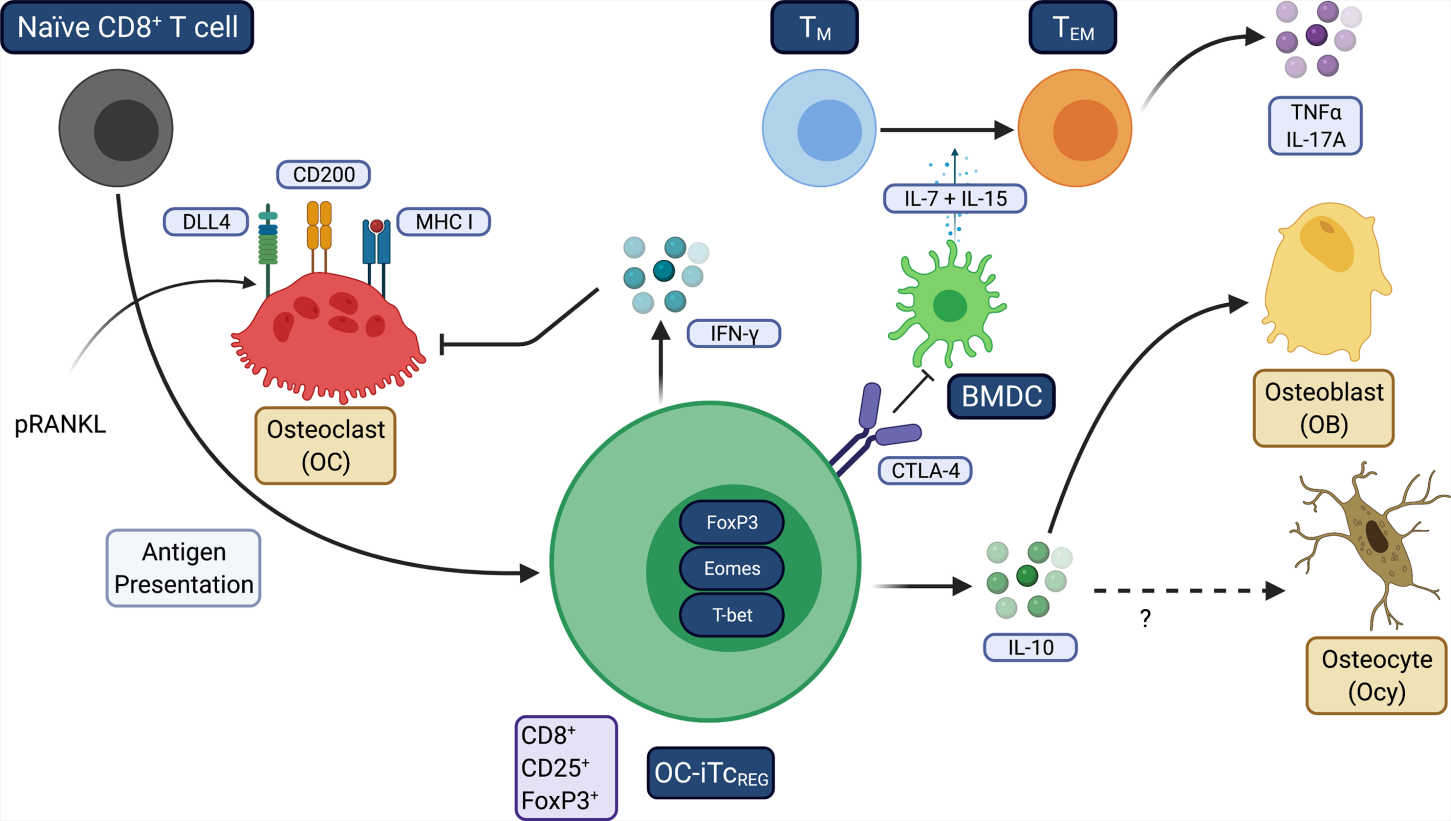
Osteoclasts induce tolerogenic Tc_REG._ This figure summarizes how OC induce Tc_REG_ to promote restore homeostasis under inflammatory conditions. OC use three signals: antigen-loaded MHC I that ligates TCR on CD8 T-cells, CD200 (a costimulation molecule that activates NF-κB) and the Notch ligand DLL4 that engages Notch1 and Notch4 on the T-cells. TNFα and/or IL-17A downregulate DLL4 expression on OC. Treatment with pRANKL leads to increased expression DLL4. Tc_REG_ secrete IFN-γ that suppress osteoclastogenesis by degrading TRAF6 and also suppress resorption by mature OC. Tc_REG_ also secrete IL-10, which is required for the bone anabolic activity but not resolution of inflammation. IL-10 may also target Ocy to improve cortical bone mass. Resolution of inflammation appears to be mediated by CTLA4 expressed on Tc_REG_.

Our laboratory discovered that OC are antigen presenting cells during bone resorption that express DLL4, CD200 and MHC I and induce naïve CD8^+^ T cells to become FoxP3^+^ regulatory T cells (Tc_REG_) (**Figure 3**). Tc_REG_ express Eomes and T-bet, have increased the surface expression of CD25 and CTLA-4, and produce IFN-γ and IL-10 (125, 126). Bone resorbing OC induce Tc_REG_ which in turn suppress bone resorption, forming a negative feedback loop (127). Tc_REG_ are also immunosuppressive in addition to regulating OC (128). Both *in vivo* induction by low dose pulsed RANKL (pRANKL) and adoptive transfer of *ex vivo* generated Tc_REG_ suppressed bone resorption, TNFα and IL-17A levels and promoted bone formation (129) to ameliorate osteoporosis in OVX mice. In unpublished studies, IL-10 directly regulate OB at the gene expression level and OVX of IL-10 deficient mice were unresponsive to the anabolic effects of pRANKL. However, Tc_REG_ retained its ability to inhibit TNFα production in T_EM_. Induction of Tc_REG_ has two facets: first, regulatory T-cells promote a tolerogenic environment by reducing the overall inflammatory burden; second, induction of regulatory T-cells is not expected to immunocompromise the host, unlike targeting inflammatory cytokines with antibodies (i.e., TNF blockade) or by JAK inhibitors. Targeting inflammatory cytokines represses both chronic and acute inflammation and thus increases risk of opportunistic infections. In contrast, inducing antigen-specific regulatory T-cells can precisely target chronic inflammation. Furthermore, while cytokine blockade may slow disease progression, regulatory T-cells promote resolution of inflammation to restore immune homeostasis (130–132). Taken together, our observations indicate that the immune system plays a fundamental role in modulating bone homeostasis, able to tip the balance either in favor of uncoupled bone resorption or bone formation.

## Conclusion and Perspectives

In this review we highlighted how T_M_ have emerged to be at the center of the pathophysiology of PMOP. These results demonstrate that E_2_ loss promotes inflammation leading to the acute phase bone erosion. We also underscore that none of the currently approved therapies for PMOP target inflammation but were intended to act directly on the BRU. The therapies also underscore that increasing bone mass may not be sufficient to reduce risk of fractures. How the proinflammatory cytokines TNFα, IL-17A and IL-1β leads to increased OC differentiation and increased resorption are well understood. In contrast, we do not have a similar level of understanding on the action of inflammation on OB and Ocy. We also highlighted how resolution of inflammation leads to increased bone formation. Additional studies are needed to understand the mechanism and the targets. In this context, better therapies will emerge from efforts to understand how Ocy sense bone quality and promote the repair process to produce bone that is resilient and less likely to fail. The correlation between bone mass (primarily by mineralization) to improving bone quality, with improved biomechanical properties needs to be further defined. Research in current decade is likely to provide new insights and mechanisms into the crosstalk. Revealing the mechanistic details on immune regulation on bone homeostasis will provide exciting new targets for therapies.

## Author Contributions

RA conceived of the manuscript. DW and RA drafted the manuscript. AP and AK created figures and helped with edits. AC-S and ES provided literature search and edits. All authors were involved in scientific discussion of the review. All authors contributed to the article and approved the submitted version.

## Funding

Research reported in this study was partially supported by National Institute of Arthritis and Musculoskeletal and Skin Disease of the NIH under Award Number RO1AR064821 and RO1AR068438. Washington University Musculoskeletal Research Core (NIH P30 AR057235) also partially supported this study.

## Conflict of Interest

The authors declare that the research was conducted in the absence of any commercial or financial relationships that could be construed as a potential conflict of interest.
